# The Effect of Omega-9 on Bone Viscoelasticity and Strength in an Ovariectomized Diet-Fed Murine Model

**DOI:** 10.3390/nu15051209

**Published:** 2023-02-28

**Authors:** Mahmoud Omer, Christopher Ngo, Hessein Ali, Nina Orlovskaya, Vee San Cheong, Amelia Ballesteros, Michael Tyrel Garner, Austin Wynn, Kari Martyniak, Fei Wei, Boyce E. Collins, Sergey N. Yarmolenko, Jackson Asiatico, Michael Kinzel, Ranajay Ghosh, Teerin Meckmongkol, Ashley Calder, Naima Dahir, Timothy A. Gilbertson, Jagannathan Sankar, Melanie Coathup

**Affiliations:** 1 Biionix Cluster, University of Central Florida, Orlando, FL 32827, USA; 2 Department of Mechanical and Aerospace Engineering, University of Central Florida, Orlando, FL 32816, USA; 3 Insigneo Institute for In Silico Medicine, University of Sheffield, Sheffield S1 3JD, UK; 4 College of Medicine, University of Central Florida, Orlando, FL 32827, USA; 5 Engineering Research Center for Revolutionizing Biomaterials, North Carolina A&T State University, Greensboro, NC 27411, USA; 6 Department of General Surgery, Nemours Children’s Hospital, Orlando, FL 32827, USA

**Keywords:** osteoporosis, omega-9, fracture, viscoelasticity, diet

## Abstract

Few studies have investigated the effect of a monosaturated diet high in ω-9 on osteoporosis. We hypothesized that omega-9 (ω-9) protects ovariectomized (OVX) mice from a decline in bone microarchitecture, tissue loss, and mechanical strength, thereby serving as a modifiable dietary intervention against osteoporotic deterioration. Female C57BL/6J mice were assigned to sham-ovariectomy, ovariectomy, or ovariectomy + estradiol treatment prior to switching their feed to a diet high in ω-9 for 12 weeks. Tibiae were evaluated using DMA, 3-point-bending, histomorphometry, and microCT. A significant decrease in lean mass (*p* = 0.05), tibial area (*p* = 0.009), and cross-sectional moment of inertia (*p* = 0.028) was measured in OVX mice compared to the control. A trend was seen where OVX bone displayed increased elastic modulus, ductility, storage modulus, and loss modulus, suggesting the ω-9 diet paradoxically increased both stiffness and viscosity. This implies beneficial alterations on the macro-structural, and micro-tissue level in OVX bone, potentially decreasing the fracture risk. Supporting this, no significant differences in ultimate, fracture, and yield stresses were measured. A diet high in ω-9 did not prevent microarchitectural deterioration, nevertheless, healthy tibial strength and resistance to fracture was maintained via mechanisms independent of bone structure/shape. Further investigation of ω-9 as a therapeutic in osteoporosis is warranted.

## 1. Introduction

Osteoporosis is a systemic, metabolic disease that progresses via gradual deterioration of the macro- and micro-architecture of skeletal tissue where the interconnecting porous system is slowly resorbed and bone mass is reduced [1,2]. As this occurs, bone porosity increases and the pores become enlarged with diversification in shape, contributing to the most important clinical complication, which is an increasingly fragile structure predisposed to low-energy insufficiency fractures [3]. It is estimated that more than 9.9 million Americans have osteoporosis and an additional 43.1 million live with low bone density [4]. As such, osteoporosis affects a substantial number of people [5], and carries a significant economic burden where the main cost driver is considered to be fracture-related treatment and associated surgical costs [6]. In the United States, ~USD 5–6.5 trillion per year is spent on disability and loss of productivity (not counting indirect costs) for the targeted treatment of osteoporosis [6]. Although most common in postmenopausal women, age-related osteoporosis is inevitable in both men and women [7,8]. In many of these cases, patients experience a significant decrease in quality of life and an increased cumulative mortality [4]. For example, hip fractures are associated with 20–40% excess mortality within 1 year post-fracture [9,10], with a higher mortality in older individuals [11], and in men (one in seven following non-hip fracture; one in three following hip fracture) more so than women (one in 11 following non-hip fracture; one in five following hip fracture) [12,13]. The greatest reduction in survival occurs within the first year post-fracture, however, a fragility fracture occurring at any fracture site, has also been associated with reduced patient survival for up to six years post-fracture [13], and even longer [14]. Patients who experienced hip fractures have a ~2.7-fold increased risk of future fractures [15,16], increasing at a rate of 4% for each year of age. The risk for fracture is 41% higher in women than men [16], with women ≥ 75 years of age experiencing a 2 year fracture risk of 25% [17]. Approximately 20% of hip fracture patients require long-term nursing home care, and only 40–60% fully regain their pre-fracture level of function, mobility, and independence [18]. Given that populations in developed nations are rapidly aging, hence an increase in the incidence of osteoporosis is inevitable. Together, these statistics indicate a clear need for improved mitigation and treatment strategies.

Several approaches for preventing osteoporosis are currently recommended. These include adopting a diet with a daily intake high in calcium-rich foods (1000 mg/day for men aged 50–70 years, and 1200 mg/day for women <51 years of age) and vitamin D (800–1000 IU/day), especially low-fat and pasteurized dairy products, fiber, protein-rich foods (e.g., meat), and regular weight-bearing and muscle-strengthening exercise [19,20,21]. The primary molecular and cellular processes involved in bone metabolism and turnover are significantly influenced by nutrients, such as fats, sugars, and proteins [22]. Although the pathogenesis of osteoporosis is multifactorial, there is emerging evidence that an unhealthy diet increases the risk of postmenopausal osteoporosis, whereas a healthy diet may decrease its occurrence [21,23]. The effect of diets high in polyunsaturated fats (PUFAs), in particular omega-3 (ω-3), and ω-6, have been widely investigated in pre-clinical and clinical studies. Results suggest that diets high in PUFA significantly increase the peak load, bone stiffness, and bone strength [24,25,26], in addition to bone mineral content (BMC), and bone mineral density (BMD) [27,28]. Diets high in ω-3 fatty acids are typically understood to support bone health, and have been associated with a reduced bone fracture risk [29,30]. However, the influence of an enriched ω-6 fatty acid diet remains inconclusive. While an increase in BMD [31,32], and a decrease in fracture risk have been reported [30,33], studies have also reported an increase in fracture risk [34,35], or no effect was measured [36]. Few studies have investigated the role of monosaturated (MUFA) ω-9 fatty acids in bone health. A recent study by our group [23], demonstrated that a 50:50 mix (saturated: unsaturated) diet high in ω-9, and relatively low in ω-3, delivered an anabolic response to bone. Our results demonstrated significantly increased bone strength and architecture when compared to the control, and also ω-6 fed animals following investigation in an 8-week high-fat diet-fed male murine model. Although all of the high-fat diets investigated in this study induced similar levels of obesity and loading in the animals, the bone response to diet varied, and the mechanism/s behind this specific ω-9 bony response remains elusive. 

Due to the ω-9-induced anabolic response measured in our previous study, here we investigated whether a high-fat diet rich in ω-9, served as a modifiable dietary intervention able to protect against the progression of post-menopausal osteoporosis. We hypothesized that a diet enriched with ω-9, and with relatively low levels of ω-3 and ω-6, would protect against a progressive osteoporotic decrease in architectural bone loss, and mechanical strength when investigated in a murine ovariectomy (OVX) model. Results were compared to OVX animals that received ‘gold standard’ estrogen supplementation. Skeletal parameters were quantified both mechanically and histologically.

## 2. Materials and Methods

All procedures were approved by the Institutional Animal Care and Use Committee at the University of Central Florida and were performed in accordance with the American Veterinary Medical Association guidelines. Female 8-week-old C57BL/6J “wild-type” mice were purchased from The Jackson Laboratory (Bar Harbor, ME, USA) and allowed to acclimatize for 2 weeks prior to ovariectomy. During this time, the mice were maintained on a 12:12-h light-dark schedule and given *ad libitum* access to an introductory, purified control diet (D07020902, consisting of 10% fat (Research Diets, Inc, New Brunswick, NJ, USA) and water. Following this two week period, mice were randomized into the experimental groups (*n* = 6), and bilateral ovariectomy surgery was performed. 

### 2.1. Ovariectomy and Dietary Intervention

Animals were anesthetized using 2% isoflurane prior to bilateral ovariectomy. Mice received subcutaneous prophylactic analgesia (carprofen, 20 mg/kg) before, and 12 h following surgical intervention. Female mice in the control group received analgesia and anesthesia, and underwent dorsal skin incision, and suturing similar to OVX animals, but without removal of the ovaries [37]. Mice were allowed to recover for 1 week post-surgery. To serve as a positive study control, and beginning on the first day following OVX, mice received either an intrascapular *s.c.* injection of 2 µg of 17β-estradiol benzoate (Sigma, St. Louis, MO, USA) dissolved in 0.1 mL sesame oil (OVX + E_2_), or the vehicle (sesame oil) only (OVX group). This hormonal regimen mimics the physiological range of estradiol (E_2_) levels found in a young adult female mouse [37]. Hormone treatment was repeated every 4 days and lasted the duration of the study. Thus, the study comprised of 3 experimental groups, namely; group 1: sham control, group 2: OVX; and group 3: OVX + E_2_.

Beginning 2 weeks post ovariectomy, the animal feed was switched to a balanced saturated: unsaturated diet high in ω-9 (D12492; Research Diets, Inc, New Brunswick, NJ, USA) and *ad libitum* over the 12-week study period (Table 1). This diet provided 60 kcal% energy from fat. Fat ingredients contained soybean oil (~13% ω-3, ~55% ω-6 and 18% ω-9) and lard (0% ω-3, ~6–10% ω-6 and ~44–47% ω-9) only, introducing ω-3, ω-6 PUFA, and ω-9 MUFAs, as well as a range of saturated fatty acids, including stearic acid, palmitic acid, oleic acid, and linoleic acid (a full description is presented in Table 2). The mean kcal fraction of each component of the diet with respect to the total kcal was calculated. The amount of ω-3, ω-6, and ω-9 was estimated (g) to determine the % contribution of ω-3, ω-6, and ω-9 within the diet.

### 2.2. Measurement of Body Weight and Body Composition

Mean body weight (g) and gain in bodyweight (g) were quantified following the introductory control diet period, and immediately prior to switching to the HFD. Weight was then measured weekly over the 12-week study period. Body composition (lean mass, and fat mass) was also measured at the end of the introductory control diet period, and prior to the mice starting the HFD diet. A Bruker minisoec LF-50 body composition analyzer (Billerica, MA, USA) was used to quantify the body composition, where data were collected immediately prior to HFD feeding, and immediately following completion of the study. Lean body mass was calculated as the difference between total body weight and body fat weight, (e.g., organs, skin, bones, body water, muscle mass). 

Mice were euthanized 12 weeks post-feed intervention, and the left and right tibia collected. All of the right tibiae were immediately plastic wrapped and stored frozen at −20 °C in preparation for, (i) dynamic mechanical testing (DMA) (storage modulus (E’), loss modulus (E’’) and loss tangent (δ)), (ii) 3-point bending analysis (ultimate stress (σu), fracture stress (σf), yield stress (σy) and elastic modulus (E)), (iii) tibial length measurement (mm) and bone area (mm^2^), (iv) cross-sectional moment of inertia (mm^4^), and, (v) micro-computed tomography (microCT) scanning (BMD (g.cm^3^), BMC (g), and BV/TV%). All mechanical testing was carried out within 2 weeks of tissue retrieval. Following dissection, all left tibiae were immediately placed in 10% buffered formaldehyde and processed for undecalcified histology. To evaluate the effect of diet alone in comparison with hormonal treatment on bone loss, samples were qualitatively assessed following toluidine blue (soft tissue) and paragon (bone) staining. The trabecular area (%), was measured in each of the groups. 

### 2.3. Dynamic Mechanical Analysis

Each tibia was analyzed under dynamic loading conditions and assessed using a dynamic materials analyzer (DMA) 242E (Artemis, Netzsch, Selb, Germany). Prior to testing, the tibiae were thawed and immersed in phosphate buffered saline (PBS) for at least 1 h at room temperature. Tibiae were orientated face down, such that the posterior aspect was subjected to three-point bending loads while under isothermal conditions. To prevent rigid body motion, 3D printed support fixtures were positioned at the proximal and distal ends of the bone, and separated at a distance of 8 mm. The central region of the (posterior) tibial midshaft, was loaded at frequencies of 0.05, 0.1, 1.0, and 10.0 Hz, and under the elastic limit of the bone. A sinewave form with a constant stress amplitude of 0.25 MPa, with a maximum (1 MPa) and minimum (0.5 MPa) stress was applied. Specimens were tested at room temperature for 60 min and the viscoelastic properties of each tibia (storage modulus E’, loss modulus E’’ and loss tangent (δ)) were obtained and results compared between the three experimental groups (*n* = 6). The storage modulus (E’) indicates how the specimen stores elastic energy while the loss modulus (E’’) is a measure of energy dissipation. The loss tangent (δ) is the phase angle between stress and strain and relates to damping. These parameters characterize the viscoelastic mechanical properties of the tibiae.

### 2.4. Three-Point Bending 

**Figure 1:**
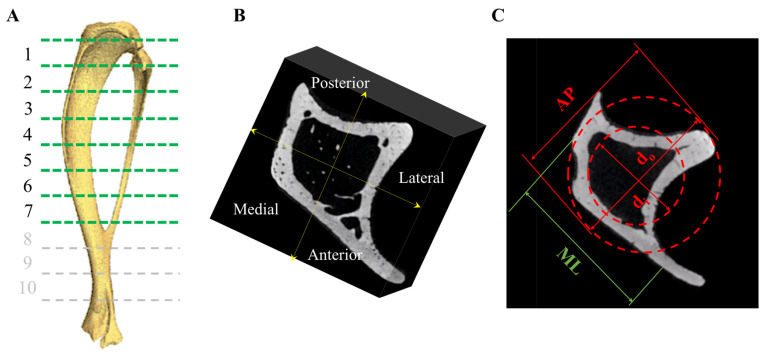
Following euthanasia, tibiae were dissected for further analyses. (**A**) Samples underwent μCT scanning and the tibia divided into 1 (proximal)–7 (tibio-fibular joint) regions along the tibial length and (**B**) in the AP and ML sectors. (**C**) A transverse μCT image of a tibia demonstrating the AP and ML directions, $d_{i}$ represents the inner diameter and $d_{o}$ the outer diameter. These values were used to calculate the biomechanical properties following 3-point testing, outer, and inner diameter, and moment of inertia.

Each tibia was loaded to failure at a displacement rate of 0.015 mm/s using a 50 N load cell, and universal testing machine (Criterion^®^ 43, Eden Prairie, MTS, Minnesota, USA). The tibiae were positioned on the fixture to lie perpendicular to the applied load, and with the anterior surface facing upwards. Fixtures were used to prevent rigid body motion, and the support bars were separated at a distance of 6 mm. Using a 3 mm diameter loading roller, a vertical force was applied to the tibial mid-shaft until structural failure. Load-displacement curves were then obtained. As an animal model was being investigated, it was acknowledged that the cross-sectional area of the tibia was non-uniform. Similar to other previously published studies [38,39], we assumed that the cross-sectional area was circular and calculated the mechanical properties including the stress, σ (Pa), in Equation (1), and elastic modulus, E (Pa), in Equation (2).

(1) $$\sigma =\frac{F*L*c_{o}}{4*I}$$

(2) $$E=\frac{FL^{3}}{d*48*I}$$

where *F* is the applied load (N), *L* = 0.006 is the span distance between the supports (m), $c_{o}$ is the outer radius of the tibia’s midshaft (m), which was measured using a calliper (Digital, Cole-Parmer, IL, U.S.), *d* is displacement (m) and *I* is the moment of inertia (m^4^) calculated using Equation (3):

(3) $$I=\frac{\pi }{4\left(c_{o}^{4}-c_{i}^{4}\right)}$$

where $c_{i}$ is the inner radius of the tibia’s midshaft (m). The inner radius of each midshaft was calculated using data obtained from the μCT scans (Figure 1C). The inner and outer diameter were calculated from four differing μCT cross-sectional regions from one bone from each experimental group. The mean values of inner-to-outer diameters from the four different cross-sections were calculated to determine the inner radius of the remaining tibia in each group. Bone area *A* (m^2^) was calculated using Equation (4):

(4) $$A=\frac{\pi }{4}\left(c_{o}^{4}-c_{i}^{4}\right)$$

The yield point was determined using an offset of 0.015 mm parallel to the linear portion at the beginning of the load-displacement plot [39]. The post yield displacement (PYD) was obtained as the displacement from the yield point to the fracture point in the load-displacement plot. Yield stress (σy), ultimate stress (σu), fracture stress (σf), elastic modulus (E), and cross-sectional moment of inertia (m^4^), were calculated. As changes in mouse bodyweight were clear, the mechanical strength parameters were adjusted for body size (ratio of body weight to tibial length) [40] (*n* = 6).

### 2.5. Histological Analysis

Following euthanasia, tibiae (*n* = 6) were prepared for undecalcified histological analysis. Samples were dehydrated in serial dilutions of alcohol, defatted, and embedded in hard grade acrylic resin (LR White, Electron Microscopy Sciences, Hatfield, UK). Using cutting and grinding techniques, longitudinal thin sections (~60 µm) were prepared through the proximal cancellous region of each tibia using a 300CP and 400CS EXAKT system (EXAKT, Germany). Sections were subsequently stained with Toluidine Blue and Paragon, which stained the soft tissue and bone, respectively. Using light microscopy and image analysis (×10 objective lens), the % trabecular bone area was measured. Five random areas (×10 objective lens) in the region immediately beneath the growth plate were imaged and % bone area was calculated using ImageJ software. Data were quantified and compared between each of the experimental groups. 

### 2.6. Scanning Electron Microscopy

To assess bone viability via the presence of osteocytes located within the lacunae, scanning electron microscopy (SEM) (Jeol, Akishima, Japan; Zeiss, Jena, Germany; Tescan, Brno, Czechia) was used to image the bone. Samples were prepared for imaging by firstly polishing the samples, and then acid-etching the surface using 9% phosphoric acid for 20 s. The samples were then washed in distilled water and 5% sodium hypochlorite, prior to further washing in distilled water, dried overnight, and sputter coated prior to observation under SEM.

### 2.7. MicroCT

Similar to our previous study [23], one tibia (*n* = 1) from each of the experimental groups was thawed in a 15 mL Eppendorf tube. MicroCT scanning was performed using a cone beam scanner (GE Phoenix Nanotom-M^TM^, Waygate Technologies, Hürth, Germany) using a 90 kV source voltage, 110 μA source current (mode 0), and a tungsten-diamond target with a 500 ms exposure time at 7–9 μm isotropic voxel resolution (depending on tibial size). Data were collected for 1080 projections over 360° (0.33° steps) with three averaged images per rotation position. The volume reconstructions were performed using Phoenix Datos software. Visualization and production of DICOM images were created using VG Studio Max (v 2.1) software. Using MATLAB 2018A (The MathWorks Inc., Natick, MA, USA), a volume of interest (VOI) was selected immediately beneath the growth plate. Bone mineral density (BMD), bone mineral content (BMC), and bone volume to total volume fraction (BV/TV%) were calculated in the anteroposterior and medio-lateral sectors in 1 (proximal)–7 (tibio-fibular joint) equi-distant regions at 80% of the tibial length below the growth plate, along the tibia in each group (Figure 1A,B) [41]. Density expressed as mg/cm^3^ of hydroxyapatite was determined using calibration phantoms of 0, 50, 200, 800, and 1200 mgHA/cm^3^. Three-dimensional Slicer software (v4.11.20210226; Brigham and Women’s Hospital and Massachusetts Institute of Technology, Boston, MA, USA) was used to create 3D models of the trabecular network within the proximal tibia. As *n* = 1, the analysis was conducted by treating all of the longitudinal sections as individual data points (Supplementary Tables S10–S13). 

### 2.8. Statistical Analysis 

Analysis of the data was performed using SPSS software (v25; SPSS, Chicago, IL, USA). Data obtained were nonparametric and the Mann–Whitney U-test was used for statistical comparison between experimental groups. The Kruskal–Wallis test with a post hoc Mann–Whitney *U*-test was used to compare data longitudinally and over the different time points within one experimental group. *p* values < 0.05 were considered significant. Means are presented with standard error (SE) values.

## 3. Results

### 3.1. Body Weight and Body Weight Gain

**Figure 2:**
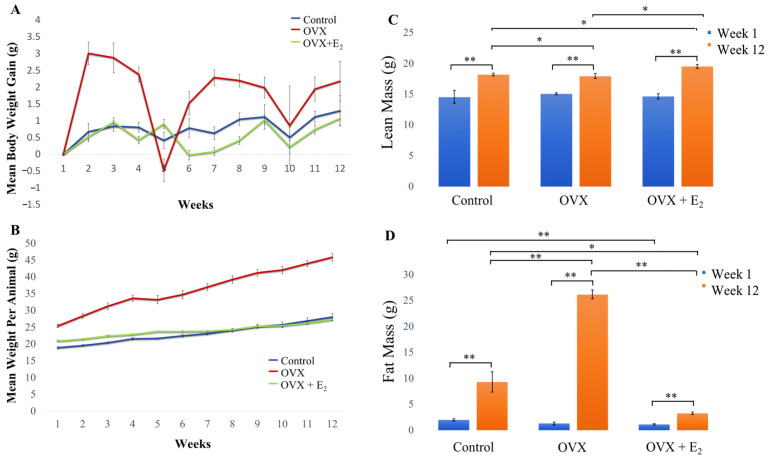
(**A**) Mean body weight per animal group (g), (**B**) mean body weight gain per week (g) in each group over the 12-week study period. (**C**) Changes in lean body mass and (**D**) fat mass. No significant difference was measured when water mass was compared between groups [data not shown]. All animals were fed a HFD diet. *n* = 6 per group. Data expressed as mean ± SE. * *p* < 0.05, ** *p* < 0.01.

All animals remained healthy throughout the duration of the study. The mean body weight per animal and weight gain in each of the groups and over the 12-week study duration is shown in Figure 2. Starting body weight, final body weight, and total cumulative gain in body weight are presented in Table 3. 

The mean body weight of animals in the control and OVX + E_2_ groups, gradually and similarly, increased over time. However, a significant increase in body weight and an overall gain in weight was observed in OVX animals throughout the 12-week period, when compared with both the control and OVX +E_2_ groups (detailed results are presented in Table 4). The mean values calculated for body weight and weight gain (g) in each group, and over each week of the study, are presented in Supplementary Tables S1 and S2.

### 3.2. Body Composition

Our findings revealed that a significant increase in lean mass was measured in each of the groups when week 1 data was longitudinally compared with week 12 (*p* < 0.01 in all groups) (Figure 2C). At the beginning of the study (week 1), no significant differences in lean mass were measured when each of the three experimental groups were compared. However, by week 12, a significant increase in lean mass was measured in the OVX E_2_ group (19.55 ± 0.30 g) when compared with the sham control (18.23 ± 0.22 g) and OVX (17.98 ± 0.41 g) groups (*p* < 0.05 in both cases). Further, significantly increased lean mass was measured in the control group when compared to the OVX animals (*p* < 0.05) at this 12-week time point. When levels of fat mass were examined, no significant difference in adiposity was found when each of the groups were compared at the beginning of the study. However, results demonstrated that adiposity significantly increased in animals in all three groups when week 1 was longitudinally compared with week 12 (Figure 2D). By week 12, a significant increase in fat mass was measured in the OVX group (26.22 ± 0.28 g), when compared to the OVX + E_2_ (3.29 ± 0.27 g) and control (9.33 ± 1.98 g) groups. As such, fat mass was measured to significantly increase ~10-fold in OVX animals when compared to the OVX + E_2_ group (*p* < 0.01) and ~2.5-fold when compared to control animals (*p* < 0.01). Finally, significantly decreased adiposity was measured in the OVX + E_2_ group of animals when compared to the control group (*p* < 0.05) at the 12-week timepoint. No other significant differences between lean and fat mass in each group were found.

### 3.3. Dynamic Mechanical Analysis (DMA)

**Figure 3:**
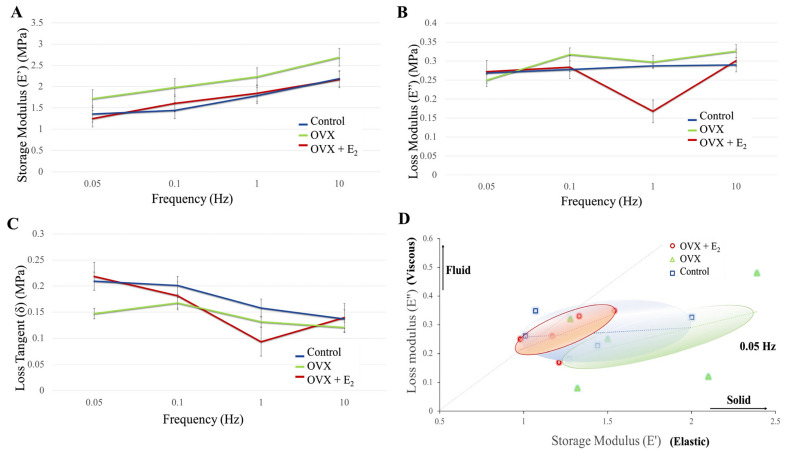
(**A**) Storage modulus, (**B**) loss modulus, (**C**) loss tangent in each of the diet groups. (**D**) A plot of the loss modulus versus storage modulus. An increased elastic and solid component is demonstrated in the OVX group when compared with the control and OVX + E_2_ groups (*n* = 6).

When storage modulus E’ (elastic energy) was examined in each group, and at increasing frequencies of 0.05, 0.1, 1.0, and 10.0 Hz, a trend was seen where E’ increased with increasing frequency (Figure 3A). It is generally accepted that within the lower frequency range (e.g., 0.05 Hz), there is greater movement of the organic component of bone, with less movement of this fraction as the frequency increases. Although a trend of a higher E’, and thus elastic component was observed in the OVX group at each frequency (0.05 Hz = 1.72 ± 0.22 MPa; 0.1 Hz = 1.98 ± 0.25 MPa; 1.0 Hz = 2.23 ± 0.24 MPa; and 10.0 Hz = 2.69 ± 0.33 MPa), no significant differences were found when compared to the control (0.05 Hz = 1.36 ± 0.18 MPa; 0.1 Hz = 1.44 ± 0.14 MPa; 1.0 Hz = 1.79 ± 0.18 MPa; and 10.0 Hz = 2.19 ± 0.23 MPa) and OVX + E_2_ (0.05 Hz = 1.25 ± 0.09 MPa; 0.1 Hz = 1.61 ± 0.10 MPa; 1.0 Hz = 1.84 ± 0.12 MPa; and 10 Hz = 2.17 ± 0.13 MPa) groups. Similarly, the loss modulus (energy dissipation) measured in the OVX group was higher when compared with the control and OVX + E_2_ groups (except at 0.05 Hz), however, no significant differences were found (Figure 3B). Changes in the loss tangent (damping) were also observed between groups (Figure 3C). At the lower frequency and stress levels of 0.05 Hz, the loss tangent reduced in the OVX group (0.15 ± 0.04) compared to the control (0.21 ± 0.04) and OVX + E_2_ (0.22 ± 0.02) groups. However, when investigated at 1.0 Hz, the lowest loss tangent was measured in the OVX + E_2_ group (0.09 ± 0.03), when compared with the control (0.16 ± 0.01) and OVX samples (0.13 ± 0.03). At the higher frequency of 10 Hz, the loss modulus was found to be similar in each of the groups. Results demonstrated that on the nano- and micro-scale, bone tissue in the OVX + E_2_ group possessed a greater viscous component when compared to the OVX group. Moreover, in the OVX group, the stiffer ‘solid’ component of bone appeared more pronounced (Figure 3D).

### 3.4. Tibial Structural Parameters

Analysis of the structural morphometric parameters measured following the 12-week study period in the control, OVX, and OVX + E_2_ groups are presented in Table 5. Our results showed that total bone area was significantly lower in the OVX group (0.84 ± 0.01 mm^2^) when compared with the control group (1.10 ± 0.04 mm^2^, *p* = 0.009). No significant difference was found when the OVX + E_2_ (1.13 ± 0.26 × 10^−3^ mm^2^) and control groups were compared. A trend was seen where the mean outer diameter decreased in the OVX group (1.37 ± 0.014 mm) when compared with the control group (1.46 ± 0.032 mm, *p* = 0.014) and OVX + E_2_ group (1.39 ± 0.075 mm), however, no significant differences were found. The decrease in cortical thickness observed in the OVX group was further confirmed as the inner diameter was also significantly highest in this group (0.90 ± 0.009 mm) when compared to the OVX + E_2_ group (0.72 ± 0.03 mm, *p* = 0.009). A significantly decreased inner diameter was measured in the OVX + E_2_ group when compared with the control group (0.86 ± 0.19 mm, *p* = 0.028), suggesting the cortices were thickest in the OVX + E_2_ group when compared with all other groups. The cross-sectional moment of inertia was lowest in the OVX group (0.14 ± 0.006 mm^4^), and significantly lower than the control group (0.20 ± 0.016 mm^4^, *p* = 0.028). 

### 3.5. Three-Point-Bending

**Figure 4:**
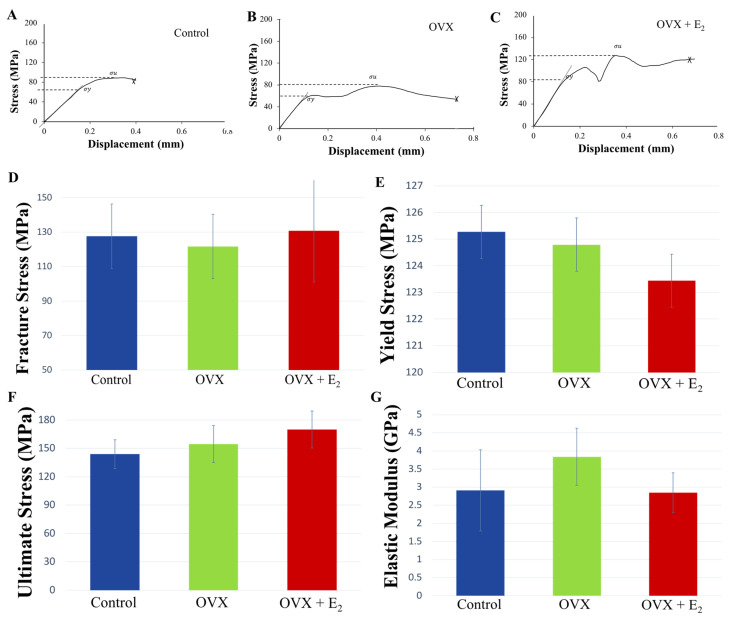
Representative stress-displacement deformation plots for tibiae in the (**A**) control, (**B**) OVX, and (**C**) OVX + E_2_ groups. “Pop-in” events were present, and were attributed to either the progression of existing microcracks present within the bone structure or the initiation of new microcracks, which ultimately culminated in whole bone fracture. The mechanical properties of (**D**) fracture strength $\sigma_{f}$, (**E**) yield stress $\sigma_{y}$, (**F**) ultimate stress $\sigma_{u}$, and (**G**) elastic modulus *E*, at the tibial mid-point in the control, OVX, and OVX +E_2_ groups (*n* = 6). Means ± SE are presented in Supplementary Table S3.

Following the dynamic loading of the tibiae under three-point bending and until failure, the mechanical properties of yield stress $\sigma_{y}$, ultimate stress $\sigma_{u}$, strength $\sigma_{f},$ and Young’s modulus *E*, varied between tibiae in each of the experimental groups. Following normalization of data to body size (ratio of body weight to tibial length), a trend was seen where animals in the OVX group displayed lower fracture, and ultimate stresses when compared to OVX + E_2_ animals. However, no significant differences were found (Figure 4). Notably, fracture strength was highest in the OVX + E_2_ group of animals (130.66 ± 13.16 MPa), and lowest in the OVX group (121.63 ± 10.05 MPa). Conversely, the elastic modulus was higher in the OVX group (4.63 ± 0.11 GPa), indicating a stiffer bone structure, and lowest in the OVX + E_2_ (2.85 ± 0.55 GPa) group of animals. The PYD is the displacement from the yield point to the fracture point, and is a measure of ductility. Higher values are considered to reflect ‘ductile’ bone, which may sustain a lot of damage before fracture [42]. Lower PYD values indicate that bone is more ‘brittle’ in nature. Our results showed that the highest PYD was measured in the OVX + E_2_ group (0.49 ± 0.17 m), followed by the OVX (0.47 ± 0.17 m) and control (0.28 ± 0.11 m) groups. No significant differences were found. The unadjusted non-normalized biomechanical properties of each of the groups are presented in Supplementary Figure S1 and Tables S3–S8. 

### 3.6. Histological, Scanning Electron Microscopy, and microCT Analyses

**Figure 5:**
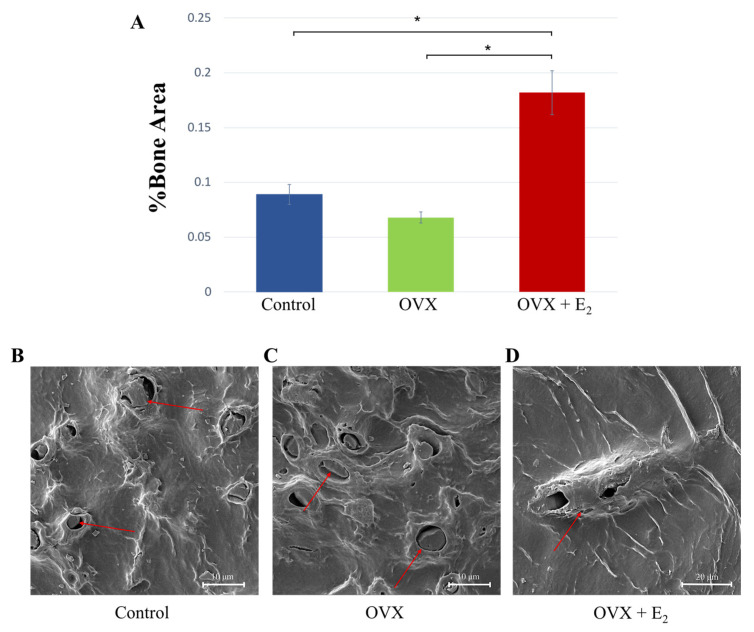
(**A**) %Trabecular bone was measured in each of the groups and the results showed a significant increase in the OVX + E_2_ group * *p* < 0.05. (**B**–**D**) Scanning electron micrographs showing osteocytes within the lacunae (red arrows); an indication of bone viability.

Quantitative assessment of %trabecular bone area showed significantly increased bone in the OVX + E_2_ group (0.182 ± 0.024%), when compared with both the control group (0.089 ± 0.009%) and OVX group (0.068 ± 0.005%; *p* < 0.05 in both cases) (Figure 5A). Samples were assessed for disparity in osteoclastic activity; however, no obvious and heightened activity was noted in the OVX group when compared with the control and OVX + E_2_ samples. Analysis using SEM showed the presence of osteocytes within lacunae in all samples, indicating similar levels of bone viability in each of the groups (Figure 5B–D).

**Figure 6:**
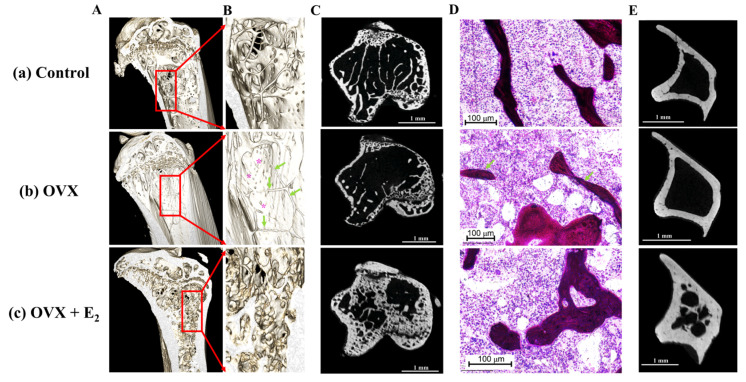
(**A**) Representative reconstructed 3D models following μCT scanning of (**a**) control, (**b**) OVX and, (**c**) OVX + E_2_ experimental groups. (**B**) (**a**–**c**) Increased magnification reveals substantial loss of bone tissue in the OVX group despite ingestion of a high fat diet enriched with ω-9. Green arrows indicate the presence of few, and thin trabeculae within the proximal tibia, exposing the cortex behind (purple asterisks). The reduction in bone tissue is also displayed in the μCT scans of the proximal tibia shown in (**C**) (**b**) and when compared to the control (**C**) (a) and OVX + E_2_ animals (**C**) (**c**). (**D**) Histological analysis revealed fewer and thinner trabeculae (green arrows) in the OVX group of animals. (**E**) Notably, an increased cortical thickness was observed in the OVX + E_2_ group when compared to both the control and OVX samples.

These results were supported following a qualitative analysis of the 3D reconstructed models and μCT images. Here, results showed substantial trabecular bone loss in the proximal region of the tibiae in the OVX group when compared to the control samples (Figure 6A–C). The anabolic effect of estradiol administration was evident via the increase observed in the size and thickness of the trabeculae when compared with the control group. Its effect was also evidenced by a thicker cortical bone structure in the transverse plane, and when compared to the control tibiae (Figure 6E).

Levels of BMD, BMC, and BVTV were assessed in one tibia from each experimental group (Supplementary Figure S2, and Tables S9–S11). Tibiae were transversely divided into seven blocks where block 1 represented the proximal region of bone, and block 7, the most distal. Bone mineral content, and BV/TV gradually increased in the proximal to distal direction in each group, while conversely, BMD gradually decreased in the proximal to distal direction. Higher BMD, BMC, and BV/TV levels were measured in the OVX + E_2_ tibiae, with lowest values displayed in OVX bone. When these parameters were compared in the antero-posterior, medio-lateral planes along the tibia (Supplementary Table S12), increased BMC, BMD, and BVTV was found in the OVX + E_2_ groups, with similar levels observed when the OVX and control tibiae were compared (Table 6).

## 4. Discussion

Presently, 8.5% of people worldwide (617 million) are elderly (≥ 65 years), and this number is forecast to increase to ~17% by 2050 (1.6 billion) [43]. As such, the prevalence of osteoporosis and incidence of fragility fractures will also undoubtedly increase [44]. Historically, osteoporosis was studied in the context of endocrine dysfunction, low estrogen, and vitamin D. However, in recent years it has been discovered that osteoporosis is multifactorial with causes stemming from immunology, the gut flora, diet, and cellular senescence among others [45]. However, the exact mechanisms remain unknown. The importance of diet on governing the development and progression of osteoporosis may be significant, but has not been fully discerned, and the role of ω-9 as a potential disease mediator has not been largely explored. Omega-9 fatty acids may work individually, additively, or synergistically as precursors and essential factors within metabolic pathways [46]. Hu et al. [47] showed that osteoporosis was regulated via 13 related metabolism pathways, including via MUFA palmitoleic acid, and therefore ω-9 fatty acids may actively contribute to regulating membrane fluidity, cell structure, and subsequent disease pathogenesis. The aim of this study was to investigate whether ovariectomized mice fed a diet high in ω-9 fatty acids, were protected against the progression of post-menopausal osteoporosis; thereby potentially serving as a modifiable dietary intervention against osteoporotic deterioration. Our rationale was based on the literature [34,48,49,50], and our previous study [23] where this targeted high fat diet induced a significant and anabolic bone response; increasing tibial viscosity, bone area, trabecular thickness, and ultimate strength, while also reducing microcrack damage in a male, murine high-fat diet-fed model. Here, our findings revealed that despite the ingestion of a diet high in ω-9 for 12 weeks following ovariectomy, a significant decrease in cortical, and trabecular area, and architecture was found when compared to the control and OVX + E_2_ groups. However, and remarkably, when the data was normalized to body weight and tibial length, no significant decrease in yield, ultimate, and fracture strength were determined when compared to the sham-operated control group. Along with other published studies [51,52,53], our results also confirmed that the progression of osteoporosis can be counteracted by estrogen treatment. Therefore, these results support our hypothesis in part.

Estradiol is a major circulating estrogen, and estrogens are known to regulate many physiological functions (e.g., reproduction, inflammation, bone formation, energy expenditure, and food intake) [52]. While increasing estrogen levels (*via* ERα [54,55]) have been associated with decreased eating [51], our results showed no significant difference in food and water uptake when each of the groups were compared [data not shown]. Animals in the OVX + E_2_ group exhibited a reduced calorific intake when compared with control and OVX animals however, no significant differences were found. Body compositional results showed that although all animals were fed a high fat diet rich in ω-9, the OVX animals (with reduced E_2_ levels) gained significantly more body weight via increased fat mass. Estrogens synergized with adipose tissue genes, and estrogen loss results in increased total adipose tissue mass [51]. As such, and following ovariectomy, this increase in fat mass was not unexpected [53] as ovulating females are generally protected from diet-induced obesity, and maintain higher energy expenditure [56], likely via upregulation of aromatase [57], the enzyme pivotal in synthesizing estrogens.

The mechanical properties of cortical bone can be characterized as: (1) an initial elastic domain where the material deforms in a reversible fashion, (2) a post-yield domain where irreversible strains and damage are produced, and (3) a fracture zone where a macrocrack or “pop-in” event is formed [42]. As such, the visco- (type-I collagen/proteins/water) elastic (hydroxyapatite (HA)) properties [58,59] of bone are of high importance in its mechanical response to load. For example, the energy absorptive properties of cancellous bone play a pivotal role in protecting bone and articular cartilage during loading [60], and falls leading to hip fracture are a high-speed event, where the dissipation of energy by bone will depend in part, on its viscoelastic properties (i.e., mass, stiffness, and damping) [61,62,63]. However, ultimate, yield, and fracture stress, and elastic modulus (stiffness) are considered primary in controlling catastrophic and monotonic bone failure [64]. Toughness is characteristic of the ability of bone to absorb energy without fracturing, and bone with a low loss tangent, is less able to dampen incoming energies, and as such, bone that is less tough, is at a higher risk for fracture [61,65,66,67]. Following ovariectomy, bone has been commonly reported to possess a reduced viscous component [68], [69], and a significantly decreased loss tangent, elastic modulus, density, ultimate force, and stiffness [67], [69,70,71,72,73,74], thus microcrack formation and rapid propagation are more likely to occur. Notably and conversely, our findings revealed that although not significant, a strong trend was seen where the tibiae retrieved in the OVX group, displayed an increased elastic modulus, and storage modulus, indicating that on the macro-structural level, and on the micro-tissue level, respectively, bone was in fact, stiffer than the control, and OVX + E_2_ groups. Previous studies have reported a correlation between bone strength and stiffness [75,76,77], where bone will adaptively stiffen to prevent fracture [78]. In support of this, we measured no significant decline in ultimate, fracture, or yield strength in the OVX group when compared with the control, and OVX + E_2_ animals. Therefore, the results from this study suggest that the high fat diet rich in ω-9 fatty acids, may have delivered an advantageous response, in that the stiffer OVX bone structure may contribute to a superior structure for protection against fracture either despite of, or to compensate for, the substantial loss in OVX bone tissue observed.

Our findings also revealed an increased PYD and loss modulus in the OVX group when compared with control mice, indicating that on the macro-structural level and on the micro-tissue level, respectively, OVX bone possessed increased ductility, and viscous energy loss. This increase would deliver enhanced compliance, high toughness, and ultimately an OVX structure that retards fracture propagation, reducing the risk of monotonic fracture [61,65,66,67]. Together, these results suggest that a diet high in ω-9, promoted both a stiffer and a more fluid component within the OVX bone tissue structure. This may have in turn, increased its overarching structural strength; a concept supported by our results which showed no significant difference in ultimate, yield, and fracture stress when compared with control and OVX + E_2_ animals.

Unexpectedly, a significant decrease in cross-sectional moment of inertia was measured in the OVX group when compared to control animals. The loss of a substantial volume of trabecular bone tissue, and thinner cortices, as measured in the OVX group, has traditionally been reported to lead to structural adaptation. Typically, a countermeasure response, where bone adjusts, and increases its cross-sectional moment of inertia, is initiated in order to elicit a more mechanically robust structure [79,80,81]. This compensational response delivered via periosteal apposition (thereby delivering an increased outer cortical diameter), combined with endosteal resorption (which increases the inner cortical diameter), may be effective in positioning the tubular bone structure further from the neutral axis. This adaptive response would distribute forces over a larger area, thereby increasing resistance to stresses and strains, thus reducing the risk of fracture [79,80,81]. This would also theoretically be at the expense of ductility but in turn, offers greater resistance to fatigue. Such structural adaptations to PUFA, and high saturated fat diet-induced osteoporosis has previously been reported following 8 weeks of feeding [23]. However, and although substantial bone loss was measured in the OVX group, no adaptive, counter-response in tibial structure was observed, but conversely a significant decrease in cross-sectional moment of inertia was measured when compared with control animals. Despite this and remarkably, OVX ultimate, fracture, and yield strength were maintained at levels similar to the control tibiae. These results suggest that the significant changes in tibial geometric, cross-sectional structure, and tissue volume shown in the OVX group, did not appear to influence the overarching mechanical properties of OVX bone. In contrast to our findings, Jimenez-Palomar and colleagues [74] reported that OVX bone micro-beams displayed a lower elastic modulus, compared to healthy samples. However, and similar to our study, an increased strain to failure (ductility) was measured when compared to healthy control samples, with no differences in bone strength measured. The authors speculated that the increased ductility provided enhanced toughness, which maintained bone strength, and that degradation of the organic material in osteoporosis is responsible for the resultant changes in mechanical properties. To this end, alterations in bone macro-structure have traditionally been considered the primary driver of osteoporotic-induced bone fragility. However, more recently, advances in our understanding revealed bone mass, spatial distribution/shape, microarchitecture, and also the intrinsic and extrinsic characteristics of the organic and inorganic matrix are of high significance [82]. Thus, osteoporotic fracture risk is governed by the complex and likely synergistic interaction of these parameters, as well as progression of the disease.

Notably, alterations in the organic matrix alter the mechanical performance of bone. The mineral phase of bone is largely composed of HA, while type-I collagen constitutes over 90% of the organic phase [58,59]. Water stabilizes the collagen triple helix via hydrogen bonding, binds to crystal surfaces for ion exchange, aiding apatite orientation and biomineralization [83], and binds extrafibrillar non-collagenous proteins, such as bone sialoprotein, osteocalcin (OCN), and osteopontin (OPN) [84,85]; thereby controlling the viscous properties of bone, delivering ductility or plasticity, and influencing the fracture properties of bone [59,86]. Further, protein networks store energy as well as dissipate large amounts of energy, thereby delivering cohesion and toughness properties to bone, and alterations in their chemistry have been reported in osteoporosis [87,88]. Changes in the mineral-organic interface due to osteoporosis have been shown to result in an increase in the stiffness and cross-linking characteristics of collagen fibers and protein chains [74]. Further, collagen fibril deformation at low strain rates, reduced fibril plasticity at high strain rates, and alterations to the intra/extrafibrillar structure have been reported in osteoporotic bone; properties that reduce the quality of the organic matrix [89]. Together, the accumulation of nonenzymatic glycation end-products, stiffening of the collagen network, as well as compositional changes in collagen, such as the α1 to α2 chains, have been reported to increase the fracture risk [90,91,92], and potentially ultimately failing via delamination of mineralized collagen [93]. We cautiously speculate that it is conceivable that the decrease in elastic modulus reported in osteoporotic bone may suggest that the stress transfer between protein molecules becomes inefficient, due to the previously reported changes in cross-linking density, thereby increasing deformation and failure within this organic component of bone [74]. If this is the case, in our present study, OVX bone was in fact stiffer. As such, the mineral portion of bone matrix likely increased, and this may have limited the interfibrillar motion of collagen, and in parallel, augmented the mechanical stability of OVX bone [68]. Paradoxically, our results also supported an increase in ductility and the viscous fraction within the organic component of bone, and the reasons for this remain elusive. High dietary levels of MUFAs have been associated with increased circulating OCN and OPN [50], which if in bone, may effect bone viscosity. Nevertheless, a high fat diet enriched with ω-9 fatty acids augmented bone strength and this may have occurred via augmentation of the organic component of bone, possibly by producing changes in cellular metabolic activity, structure, and/or cell function, in addition to potentially promoting osteoblast function [94].

Several study limitations are acknowledged. First, BMD, BMC, and BVTV were measured in only one animal per group. As the level of mineralization renders bone its stiffness, further investigations are needed to thoroughly assess alterations, and any correlations in bone mineral content, viscoelasticity, and bone fracture in response to ω-9, and following ovariectomy. Although an *n* = 1, our data showed that the OVX bone and control tibiae presented with similar levels of BMD and BMC. Second, this study did not assess levels of crystallinity, crystal properties, osteogenic, and non-collagenous protein levels, or collagen content and quality; all critical mediators of bone strength and risk of fracture. Finally, routine clinical laboratory data that would deliver further information on the biochemical changes that had occurred (e.g., glucose, triglycerides, creatine, OCN, OPN) were not investigated in this study.

## 5. Conclusions

In conclusion, and to the best of our knowledge, there are few studies that have investigated the effect of a diet high in ω-9 on bone health and osteoporosis. This study provides first evidence of the contribution of ω-9 in bone health and during post-menopausal osteoporosis. While studies in the literature report that osteoporotic bone exhibits decreased stiffness, storage modulus, loss modulus, and viscosity, increasing their cross-sectional moment of inertia to mechanically compensate for tissue loss, our study demonstrated that these attributes were not observed following administration of a diet high in ω-9 fatty acids, and over a post-ovariectomy 12-week study period. Notably, the diet did not prevent osteoporotic deterioration of the trabecular and cortical bone structure, nevertheless healthy overarching tibial strength and resistance to fracture was maintained. The ω-9 diet delivered subtle changes to the material properties of bone, and the unique skeletal properties bestowed by the ω-9 diet may be due to changes in metabolic activity within the organic component of bone. Many questions remain, and further investigation of ω-9 as a modifiable dietary intervention against osteoporotic deterioration is warranted.

## Figures and Tables

**Table 1:** The experimental murine diet (D12492) and the estimated contribution of the essential ω-3, ω-6, and ω-6 fatty acids within the diet. The HFD diet was composed of 9.3% PUFA (soybean) and 91.7% of a monounsaturated and saturated mix. * The control diet was not investigated in this study and is included for reference purposes only.

Diet Type	Diet	Fat Ratio: (Unsaturated: Saturated)	Total Fat (kcal%)	ω-3%	ω-6%	ω-9%
Control Diet *	D07020902	1:1	10	2.8	31.6	30.7
High Fat Diet	D12492	1:1	60	1.25	14.15	44.2

**Table 2:** Rodent diet with 10 or 60 kcal% fat and modification to fat sources and fat level. * The control diet was not investigated in this study and is included for reference purposes only. ** Vitamin mix (V1001): vitamin A acetate (500,000 IU/g), vitamin D3 (100,000 IU/g), vitamin E acetate (500 IU/g), menadione sodium bisulfate (62.5% menadione), biotin (1.0%), cyanocobalamin (0.1%), folic acid (0.2 g), nicotinic acid (3 g), calcium pantothenate (1.6 g), pyridoxine-HCl (0.7 g), riboflavin (0.6 g), thiamine HCl (0.6 g) and sucrose (978.42 g).

	* Control Diet		HFD ^50:50^	
	g	kcal	g	kcal
Protein (% by weight)	19.2	20	26.2	20
Carbohydrate (% by weight)	67.3	70	26.3	20
Fat (% by weight)	4.3	10	34.9	60
Total kcal		100		100
kcal/g	3.85		5.24	
Ingredients				
Protein Casein, 80 Mesh	200	800	200	800
L-Cystine	3	12	3	12
Carbohydrate Corn Starch Maltodextrin 10 Sucrose Cellulose, BW200	500 100 100 50	2000 400 400 0	0 125 68.8 50	0 500 275 0
Lipid Soybean Oil Lard CoconutOil, Hydrogenated Safflower Oil Cocoa Butter	10 5 0 0 30	90 45 0 0 270	25 245 0 0 0	225 2205 0 0 0
Mineral Mix S10026 Dicalcium phosphate Calcium carbonate Potassium citrate, 1 H_2_O	13 5.5 16.5	0 0 0	13 5.5 16.5	0 0 0
Vitamin Mix V10001 ** Choline Bitartrate	10 2	40 0	10 2	40 0
FD&C Yellow Dye #5 FD&C Red Dye #40 FD&C Blue Dye #1	0 0 0	0 0 0	0 0 0.05	0 0 0
Total	1055	4057	773.85	4057

**Table 3:** Data is expressed as mean ± SE. Differences in starting body weight, final body weight, and the total cumulative gain in weight in each of the experimental groups are presented (*n* = 6).

Variables	Control	OVX	OVX + E_2_
Starting body weight (g)	18.92 ± 0.32	25.41 ± 0.49	20.96 ± 0.23
Final body weight (g)	28.05 ± 1.10	45.09 ± 1.14	27.32 ± 0.35
Total cumulative gain in body weight (g)	9.13	20.49	6.36

**Table 4:** A statistical comparison of body weight (BW) and weight gain (WG) in each of the groups. *p* < 0.05 were considered significant.

Study Week	Body Weight (BW)	Group Comparison	*p*-Value * *p* < 0.05	Weight Gain (WG)	*p*-Value * *p* < 0.05
1	BW	Control vs. OVX Control vs. OVX + E_2_ OVX vs. OVX + E_2_	0.004 * 0.002 * 0.002 *	WG	- - -
2	BW	Control vs. OVX Control vs. OVX + E_2_ OVX vs. OVX + E_2_	0.004 * 0.003 * 0.002 *	WG	0.004 * 0.366 0.0028 *
3	BW	Control vs. OVX Control vs. OVX + E_2_ OVX vs. OVX + E_2_	0.004 * 0.003 * 0.002 *	WG	0.004 * 0.605 0.005 *
4	BW	Control vs. OVX Control vs. OVX + E_2_ OVX vs. OVX + E_2_	0.004 * 0.010 * 0.002 *	WG	0.004 * 0.093 0.002 *
5	BW	Control vs. OVX Control vs. OVX + E_2_ OVX vs. OVX + E_2_	0.004 * 0.002 * 0.002 *	WG	0.103 0.121 0.002 *
6	BW	Control vs. OVX Control vs. OVX + E_2_ OVX vs. OVX + E_2_	0.004 * 0.028 * 0.002 *	WG	0.014 * 0.109 0.003 *
7	BW	Control vs. OVX Control vs. OVX + E_2_ OVX vs. OVX + E_2_	0.004 * 0.196 0.002 *	WG	0.004 * 0.039 0.002 *
8	BW	Control vs. OVX Control vs. OVX + E_2_ OVX vs. OVX + E_2_	0.004 * 0.796 0.002 *	WG	0.006 * 0.024 0.002 *
9	BW	Control vs. OVX Control vs. OVX + E_2_ OVX vs. OVX + E_2_	0.004 * 0.796 0.002 *	WG	0.078 0.606 0.020 *
10	BW	Control vs. OVX Control vs. OVX + E_2_ OVX vs. OVX + E_2_	0.004 * 1.000 0.002 *	WG	0.337 0.302 0.302
11	BW	Control vs. OVX Control vs. OVX + E_2_ OVX vs. OVX + E_2_	0.004 * 0.606 0.002 *	WG	0.037 * 0.121 0.004 *
12	BW	Control vs. OVX Control vs. OVX + E_2_ OVX vs. OVX + E_2_	0.004 * 0.518 0.002 *	WG	0.200 0.897 0.078

* *p* < 0.05.

**Table 5:** The structural morphometric parameters of the control, OVX, and OVX + E_2_ tibiae. Data are presented as mean ± standard error (*n* = 6).

Tibial Variables	Control	OVX	OVX + E_2_
Total area (mm^2^)	1.10 ± 0.04	0.84 ± 0.01	1.13 ± 0.11
Outer diameter (mm)	1.46 ± 0.03	1.37 ± 0.01	1.39 ± 0.08
Inner diameter (mm)	0.86 ± 0.19	0.90 ± 0.009	0.72 ± 0.03
Moment of inertia (mm^4^)	0.20 ± 0.02	0.14 ± 0.006	0.18 ± 0.04
Length (mm)	18.82 ± 0.45	21.30 ± 0.35	19.64 ± 0.87

**Table 6:** A comparison of the mean values obtained for whole bone mineral and volume levels in the control, OVX, and OVX + E_2_ experimental groups following the 12-week study period.

Whole Tibial Bone Mineral and Volume Levels (Range)			
	Control	OVX	OVX + E_2_
BMD (g.cm^3^)	0.461 (0.310–0.673)	0.422 (0.267–0.623)	0.585 (0.390–0.844)
BMC (g)(sum)	7.327	6.749	10.010
BV/TV	0.547 (0.253–0.732)	0.490 (0.241–0.646)	0.689 (0.514–0.853)
Whole tibial bone mineral and volume levels (range)			
	Control	OVX	OVX + E_2_
BMD (g.cm^3^)	0.461 (0.310–0.673)	0.422 (0.267–0.623)	0.585 (0.390–0.844)
BMC (g)(sum)	7.327	6.749	10.010
BV/TV	0.547 (0.253–0.732)	0.490 (0.241–0.646)	0.689 (0.514–0.853)

## Data Availability

The data that support the findings of this study are available from the corresponding author, [M.C.], upon reasonable request.

## Supplementary Materials

The following supporting information can be downloaded at: https://www.mdpi.com/article/10.3390/nu15051209/s1, Figure S1: The mechanical properties of (A) fracture strength $\sigma_{f}$, (B) yield stress $\sigma_{y}$, (C) ultimate stress $\sigma_{u}$, and (D) elastic modulus *E*, at the tibial mid-point in the control, OVX, and OVX +E_2_ groups (*n* = 6) **p* < 0.05, ** *p*< 0.01; Table S1: Mean values ± standard error of body weight (g) measured in each of the groups and over the 12-week study period. Table S2: Mean values ± standard error of body weight gain (g) measured in each of the groups and over the 12-week study period. Table S3: The biomechanical strength properties at the tibial midpoint in each of the groups. Values are normalized according to body weight and tibial length. Table S4: The biomechanical strength properties at the tibial midpoint in each of the groups. Values are [non-normalized] according to body weight and tibial length. Table S5: [Unadjusted data]. *p* values obtained following the comparison of yield stress (MPa) in each of the groups. Table S6: [Unadjusted data]. *p* values obtained following the comparison of ultimate stress (MPa) in each of the groups. Table S7: [Unadjusted data]. *p* values obtained following the comparison of fracture stress (MPa) in each of the groups. Table S8: [Unadjusted data]. *p* values obtained following the comparison of elastic modulus (GPa) in each of the groups. Table S9: [Unadjusted data]. *p* values obtained following the comparison of moment of inertia in each of the groups. *denotes a significant difference. Figure S2: (A) Bone mineral content (BMC) from the proximal (block 1) to the distal (block 7) region of the tibia in each of the experimental groups. (B) A comparison of BMC in the anterior, posterior, medial, and lateral aspects of the tibia. Similar results are presented for (C,D) BMD, and (E,F) BV/TV (*n* = 1). Table S10: Mean values of BMC (g) in whole bone and per block in each of the groups. Table S11: Mean values of BMD (g.cm^3^) in whole bone and per block in each of the groups. Table S12: Mean values of BV/TV in whole bone and per block in each of the groups. Table S13: Tibial bone mineral and volume levels in the antero-posterior and medio-lateral aspects. Data obtained from one animal per group.
