# Supplementary material for: The Effect of Omega-9 on Bone Viscoelasticity and Strength in an Ovariectomized Diet-Fed Murine Model
Source: Nutrients. 2023 Feb 28;15(5):1209. doi: 10.3390/nu15051209 (PMC10005705; doi:10.3390/nu15051209)
Supplement: Supplementary file 1 [file nutrients-15-01209-s001.zip › nutrients-2186209-supplementary.pdf]

Supplementary Figures

Study Week	Control BW (mean ± SE)	OVX BW (mean ± SE)	OVX + E_2 BW (mean ± SE)
1	18.92 ± 0.32	25.41 ± 0.49	20.96 ± 0.23
2	19.60 ± 0.26	28.42 ± 0.73	21.49 ± 0.21
3	20.44 ± 0.27	31.30 ± 0.96	22.44 ± 0.26
4	21.26 ± 0.37	33.69 ± 0.90	22.87 ± 0.26
5	21.68 ± 0.19	33.23 ± 1.22	23.78 ± 0.20
6	22.47 ± 0.41	34.75 ± 1.22	23.76 ± 0.24
7	23.10 ± 0.43	37.04 ± 1.05	23.84 ± 0.22
8	24.14 ± 0.53	39.25 ± 1.13	24.26 ± 0.23
9	25.26 ± 0.65	41.23 ± 1.02	25.29 ± 0.28
10	25.77 ± 0.90	42.09 ± 1.09	25.51 ± 0.35
11	26.89 ± 0.98	44.05 ± 0.91	26.25 ± 0.35
12	28.05 ± 1.11	45.90 ± 1.14	27.32 ± 0.35

Supplementary Table S1: Mean values ± standard error of body weight (g) measured in each of the groups and over the 12-week study period.

Study Week	Control WG (mean ± SE)	OVX WG (mean ± SE)	OVX + E_2 WG (mean ± SE)
1	0.00	0.00	0.00
2	0.68 ± 0.25	3.01 ± 0.34	0.53 ± 0.15
3	0.84 ± 0.15	2.88 ± 0.44	0.96 ± 0.13
4	0.81 ± 0.15	2.38 ± 0.22	0.43 ± 0.11
5	0.42 ± 0.25	-0.47 ± 0.34	0.91 ± 0.13
6	0.79 ± 0.28	1.53 ± 0.35	-0.02 ± 0.14
7	0.63 ± 0.19	2.29 ± 0.23	0.08 ± 0.12
8	1.05 ± 0.19	2.20 ± 0.19	0.42 ± 0.12
9	1.12 ± 0.37	1.99 ± 0.31	1.02 ± 0.14
10	0.51 ± 0.30	0.86 ± 1.18	0.22 ± 0.19
11	1.12 ± 0.17	1.95 ± 0.36	0.74 ± 0.14
12	1.30 ± 0.45	2.18 ± 0.59	1.07 ± 0.20

Supplementary Table S2: Mean values ± standard error of body weight gain (g) measured in each of the groups and over the 12-week study period.

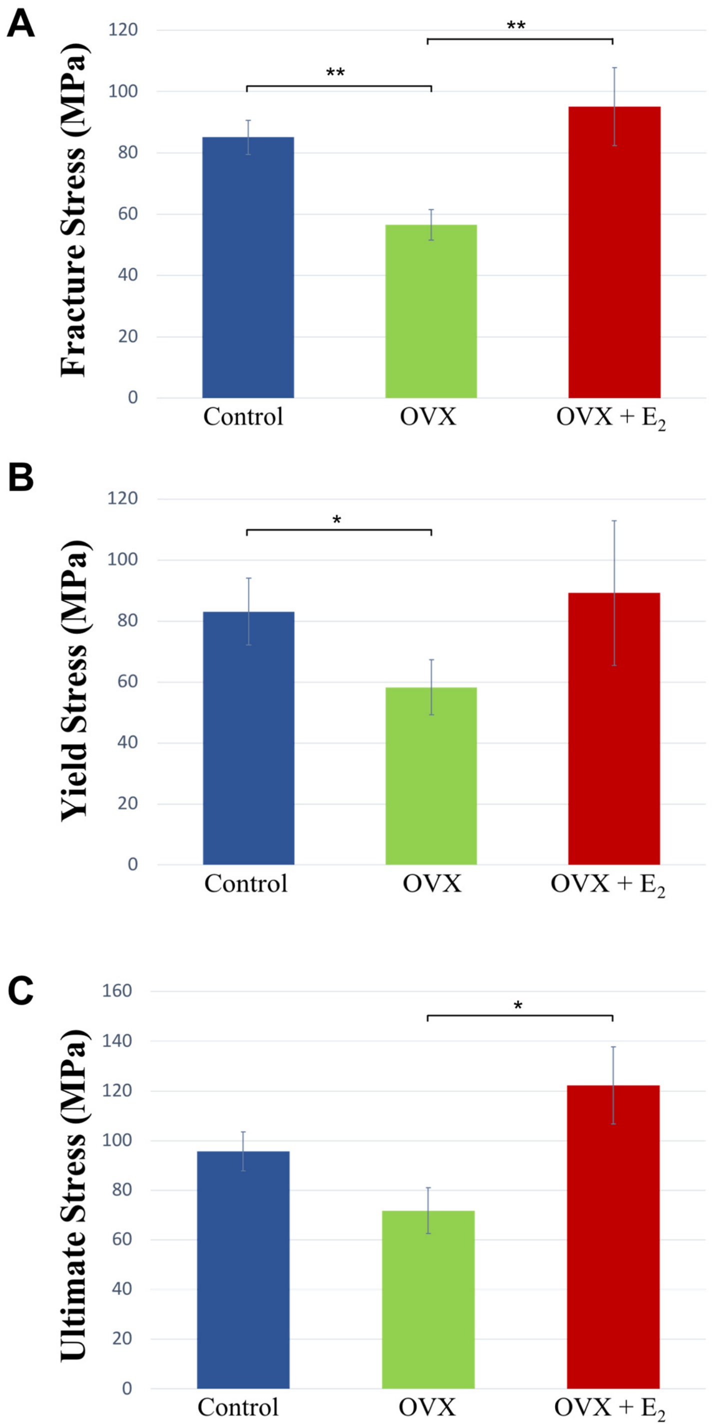

Supplementary Figure S1. The mechanical properties of [A] fracture strength σ_f, [B] yield stress σ_y, [C] ultimate stress σ_u, and [D] elastic modulus E, at the tibial mid-point in the control, OVX, and OVX +E_2 groups (n = 6) *$p < 0.05$, ** $p < 0.01$

Variables	*Control*	*OVX*	*OVX + E_2*
Yield stress $\sigma y\ (MPa)$	125.27 ± 18.73	124.79 ± 18.72	123.44 ± 29.69
Ultimate stress $\sigma u\ (MPa$	143.94 ± 15.27	154.53 ± 19.55	170.06 ± 19.45
Fracture stress $\sigma f\ (MPa)$	127.60 ± 10.66	121.63 ± 10.04	130.76 ± 29.42
Elastic modulus E (GPa)	2.91 ± 1.12	3.84 ± 0.79	2.85 ± 0.55

Supplementary Table S3: The biomechanical strength properties at the tibial midpoint in each of the groups. Values are normalized according to body weight and tibial length

Variables	*Control*	*OVX*	*OVX + E_2*
Yield stress $\sigma y\ (MPa)$	83.11 ± 10.88	58.21 ± 9.06	89.17 ± 23.67
Ultimate stress $\sigma u\ (MPa$	95.69 ± 7.89	71.73 ± 9.24	122.32 ± 15.54
Fracture stress $\sigma f\ (MPa)$	85.09 ± 5.57	56.49 ± 4.91	95.05 ± 12.68
Elastic modulus E (GPa)	1.92 ± 0.29	1.77 ± 0.36	2.06 ± 0.45

Supplementary Table S4: The biomechanical strength properties at the tibial midpoint in each of the groups. Values are [non-normalized] according to body weight and tibial length

	Control	OVX	OVX + E_2
Control			
OVX	0.754		
OVX + E2	0.917	0.754	

Supplementary Table S5: [Unadjusted data]. *p* values obtained following the comparison of yield stress (MPa) in each of the groups.

	Control	OVX	OVX + E_2
Control			
OVX	0.602		
OVX + E2	0.251	0.602	

Supplementary Table S6: [Unadjusted data]. *p* values obtained following the comparison of ultimate stress (MPa) in each of the groups.

	Control	OVX	OVX + E_2
Control			
OVX	0.754		
OVX + E2	0.917	0.465	

Supplementary Table S7: [Unadjusted data]. *p* values obtained following the comparison of fracture stress (MPa) in each of the groups.

	Control	OVX	OVX + E_2
Control			
OVX	0.251		
OVX + E2	0.917	0.251	

Supplementary Table S8: [Unadjusted data]. *p* values obtained following the comparison of elastic modulus (GPa) in each of the groups.

	Control	OVX	OVX + E_2
Control			
OVX			
OVX + E2			

Supplementary Table S9: [Unadjusted data]. *p* values obtained following the comparison of moment of inertia in each of the groups. *denotes a significant difference

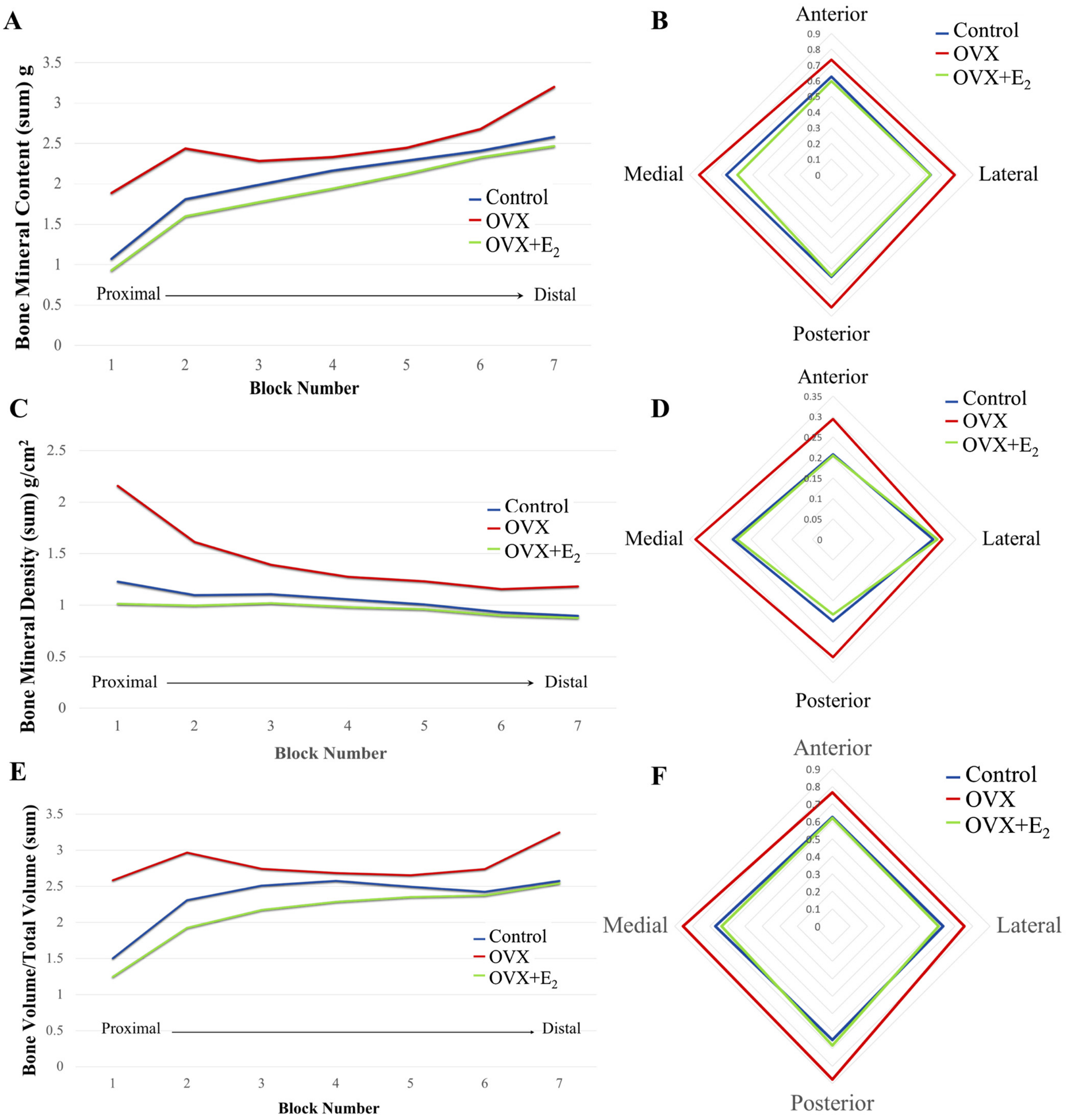

Supplementary Figure S2. [A] Bone mineral content (BMC) from the proximal (block 1) to the distal (block 7) region of the tibia in each of the experimental groups. [B] A comparison of BMC in the anterior, posterior, medial, and lateral aspects of the tibia. Similar results are presented for [C,D] BMD, and [E,F] BV/TV (*n*=1).

Variable	*Control*	*OVX*	*OVX + E_2*
Total BMC (g) whole bone	7.327	6.749	10.010
Total BMC by block			
1 (proximal)	1.228	1.014	2.159
2	1.098	0.997	1.614
3	1.108	1.020	1.392
4	1.058	0.981	1.275
5	1.007	0.969	1.232
6	0.931	0.901	1.156
7 (distal)	0.896	0.877	1.184

Supplementary Table S10. Mean values of BMC (g) in whole bone and per block in each of the groups.

Variable	*Control*	*OVX*	*OVX + E_2*
BMD ($g.cm^3$) whole bone	0.461	0.422	0.585
BMD by block			
1	0.267	0.232	0.472
2	0.452	0.399	0.609
3	0.497	0.443	0.570
4	0.542	0.485	0.583
5	0.572	0.531	0.611
6	0.603	0.582	0.670
7	0.645	0.617	0.780

Supplementary Table S11. Mean values of BMD ($g.cm^3$) in whole bone and per block in each of the groups.

Variable	*Control*	*OVX*	*OVX + E_2*
BV/TV whole bone	0.547	0.490	0.689
BV/TV by block			
1	0.375	0.311	0.646
2	0.577	0.481	0.742
3	0.627	0.543	0.685
4	0.644	0.571	0.671
5	0.624	0.587	0.663
6	0.606	0.593	0.685
7	0.644	0.635	0.812

Supplementary Table S12. Mean values of BV/TV in whole bone and per block in each of the groups.

Variables	*Control*	*OVX*	*OVX + E_2*
AP-ML tibia bone mineral and volume levels			
BMD ($g.cm^3$):			
Anterior	0.486	0.427	0.588
Lateral	0.505	0.465	0.625
Medio	0.405	0.394	0.561
Posterior	0.438	0.399	0.564
BMC (g):			
Anterior	1.994	1.931	2.819
Lateral	2.079	1.776	2.582
Medio	1.373	1.222	1.987
Posterior	1.880	1.821	2.622
BV/TV:			
Anterior	0.613	0.532	0.724
Lateral	0.576	0.507	0.711
Medio	0.480	0.468	0.659
Posterior	0.508	0.449	0.656

Supplementary Table S13: Tibial bone mineral and volume levels in the antero-posterior and medio-lateral aspects. Data obtained from 1 animal per group.
